# Supplementary material for: Quantifying the impact of early life growth adversity on later life health
Source: Commun Med (Lond). 2025 Nov 17;5:534. doi: 10.1038/s43856-025-01245-3 (PMC12749450; doi:10.1038/s43856-025-01245-3)
Supplement: Supplementary file 2 — Supplementary Information [file 43856_2025_1245_MOESM2_ESM.pdf]

Supplement to Goldman-Pham R, et al. Quantifying the impact of early life growth adversity on later life health.

Authors: Raphael Goldman-Pham et al

Contents:

Supplementary Figures 2

Supplementary Tables 4

Supplementary Figure and Tables:

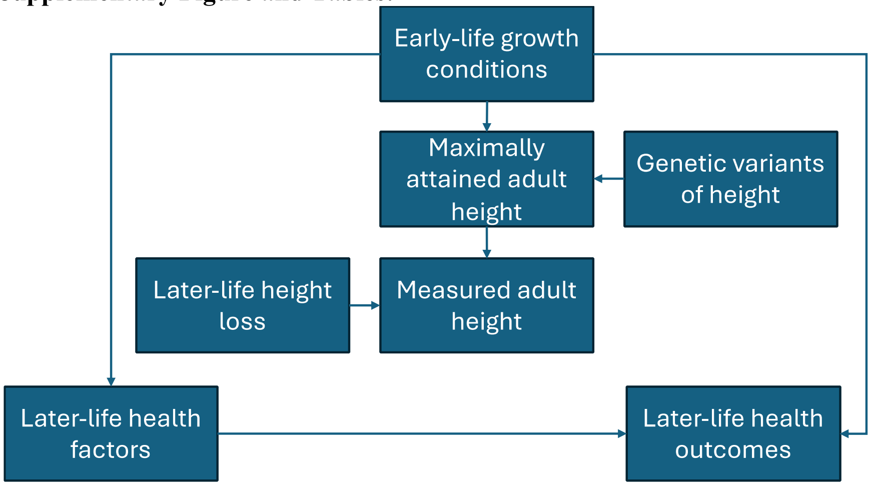

Supplementary Figure 1. A directed acyclic graph depicting hypothesized causal relationships of early-life growth conditions with late-life health outcomes.

"Later-life health factors" refers to later-life exposures/events/behaviours (e.g., adult hypertension) that may result from early-life growth adversity (e.g., prematurity) and are potentially causally related to later-life health outcomes (e.g. mortality).

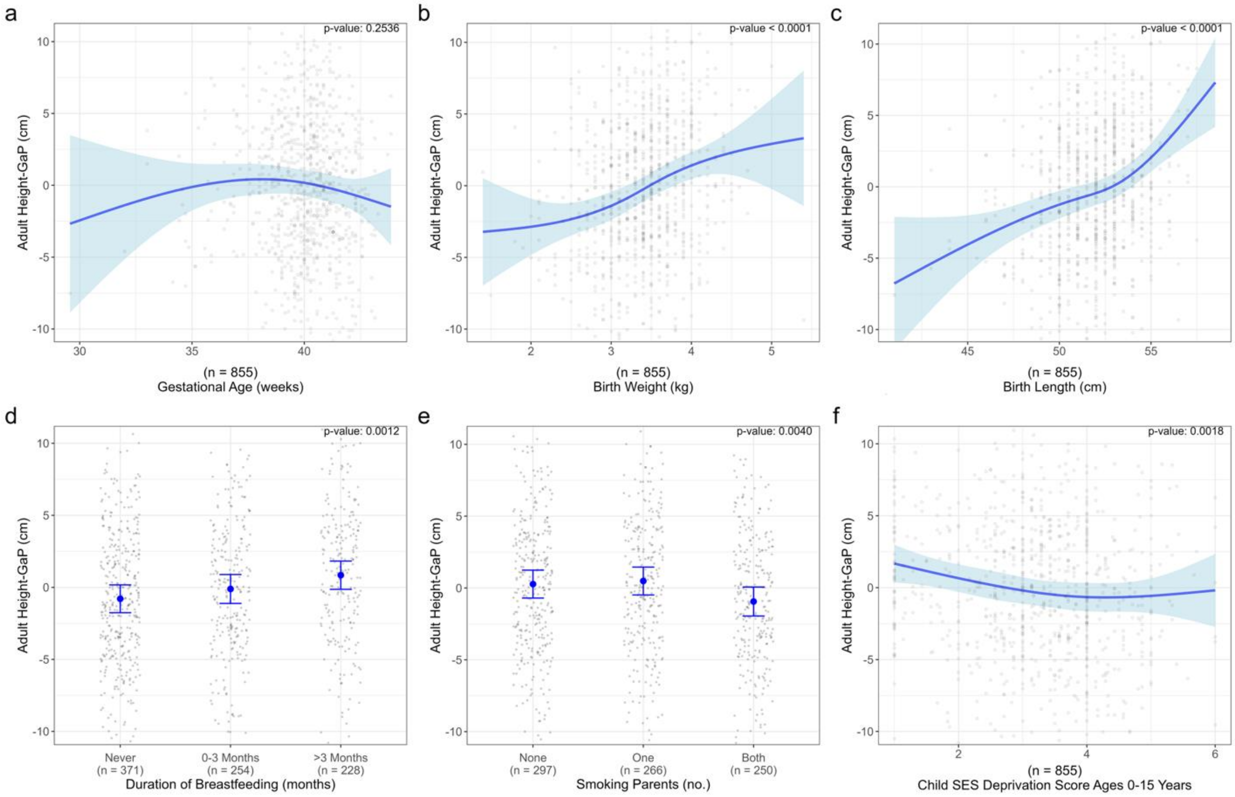

Supplementary Figure 2: Estimated marginal mean adult height-GaP with early-life growth conditions assessed in the Dunedin Multidisciplinary Health and Development Study.

a) gestational age at birth, b) birth weight, c) birth length, d) duration of breastfeeding, e) number of tobacco-smoking parents, f) socio-economic status in quintiles between ages 0-15 years. Models are adjusted for sex, age at height-GaP assessment, and genotype-predicted height. Shaded error bands (panels a-c, f) and vertical error bars (panels d, e) and represent the 95% confidence interval (precision) of the estimated mean height-GaP. Black dots indicate participant data points. P-values reflect two-sided Wald tests and are not adjusted for multiple testing. The p-value threshold to infer statistical significance for this replication analysis was set at 0.050.
Abbreviations: SES = socio-economic status

Supplementary Table 1: Characteristics of ALSPAC participants included and excluded from analyses.

	**Included**	**Excluded**
No.	4,582	11,063
Sex, no. (%)		
Female	2,579 (56.3)	1,716 (40.7)
Male	2,003 (43.7)	2,499 (59.3)
Age at height-GaP assessment, years [IQR]	24 (18, 25)	24 (18, 25)
Genotype-predicted height cm	171.9 (7.9)	173.8 (7.7) [n=4215, % missing=61.9]
Height-GaP, cm	0.0 (5.1)	NA [n=0, % missing=100.0]
Height, cm	171.9 (9.4)	170.5 (9.1) [n=1,414, % missing=87.2]
Pregnancy index of multiple deprivation quintile	2.91 (1.38)	3.40 (1.40)
Maternal smoking during pregnancy, no. (%)		
Never	3751 (87.3)	6913 (77.0)
1-4 cigarettes/day, n (%)	144 (3.4)	384 (4.3)
5-9 cigarettes/day, n (%)	126 (2.9)	510 (5.7)
10-19 cigarettes/day, n (%)	209 (4.9)	842 (9.4)
20+ cigarettes/day, n (%)	67 (1.6)	333 (3.7)
"Healthy" diet principal component during pregnancy	0.05 (0.99)	-0.03 (1.01)
Gestational age at birth, weeks	39.46 (1.83)	37.90 (6.41)
Birth length, cm	50.61 (2.13)	50.46 (2.24)
Birth weight, kg	3.43 (0.53)	3.36 (0.60)
Breastfeeding duration, no. (%)		
Never	641 (15.8)	2115 (29.9)
<1 month	580 (14.3)	1227 (17.4)
1-<3 months	623 (15.4)	1140 (16.1)
3-<6 months	601 (14.8)	819 (11.6)
6+ months	1608 (39.7)	1764 (25.0)
Childhood index of multiple deprivation ages 0-12 quintile, mean	2.77 (1.26)	3.33 (1.34)
Childhood household tobacco smoke exposure duration, no. (%)		
0 hours/week	1813 (54.2)	1736 (41.8)
>0-5 hours/week	984 (29.4)	1247 (30.0)
>5-10 hours/week	177 (5.3)	296 (7.1)
>10-20 hours/week	189 (5.6)	365 (8.8)
20+ hours/week	183 (5.5)	514 (12.4)
"Healthy" diet principal component at 38 months	0.05 (0.99)	-0.03 (1.01)
Residential outdoor $PM_{2.5}$ concentration, μg/m^3	13.26 (0.77)	13.34 (0.71)

Abbreviations: ALSPAC = Avon Longitudinal Study of Parents and Children; $PM_{2.5}$ = particulate matter with diameter less than 2.5 micrometres.

Supplementary Table 2: Characteristics of participants included in the Dunedin Multidisciplinary Health and Development Study analyses.

	**All**		**By Height-GaP Quartile**							
			**1**		**2**		**3**		**4**	
No.	855		214		214		214		213	
Sex, no. (%)										
Female	419 (49.0)		111 (51.9)		103 (48.1)		92 (43.0)		113 (53.1)	
Male	436 (51.0)		103 (48.1)		111 (51.9)		122 (57.0)		100 (46.9)	
Age at height-GaP assessment, years	26 (26, 26)		26 (26, 26)		26 (26, 26)		26 (26, 26)		26 (26, 26)	
	Male	Female	Male	Female	Male	Female	Male	Female	Male	Female
Genotype-predicted height cm	178.2 (3.5)	165.2 (3.6)	178.1 (3.5)	165.5 (3.8)	178.1 (3.5)	164.8 (3.5)	178.4 (3.5)	165.3 (3.5)	178.2 (3.3)	165.1 (3.4)
Height-GaP, cm	0.0 (5.5)	0.0 (5.1)	-6.8 (2.9)	-6.1 (2.5)	-1.8 (1.0)	-1.8 (1.00)	1.5 (1.0)	1.7 (1.0)	7.1 (3.5)	6.3 (2.8)
Height, cm	178.2 (6.5)	165.2 (6.2)	171.3 (4.8)	159.4 (4.7)	176.3 (3.4)	163.0 (3.7)	179.9 (3.5)	166.9 (3.8)	185.3 (4.7)	171.4 (4.5)
Gestational age at birth, weeks	40.0 (1.7)		40.1 (1.8)		40.1 (1.7)		40.0 (1.5)		39.9 (1.6)	
Birth length, cm	51.9 (2.3)		51.1 (2.4)		51.8 (2.3)		52.1 (2.0)		52.6 (2.2)	
Birth weight, kg	3.39 (0.52)		3.23 (0.54)		3.35 (0.51)		3.44 (0.46)		3.52 (0.51)	
Breastfeeding duration, no. (%)										
Never	371 (43.5)		108 (50.9)		100 (46.7)		86 (40.2)		77 (36.2)	
0-3 Months	254 (29.8)		58 (27.4)		66 (30.8)		70 (32.7)		60 (28.2)	
>3 Months	228 (26.7)		46 (21.7)		48 (22.4)		58 (27.1)		76 (35.7)	
Childhood (ages 0-15 years) socio-economic deprivation score	3.19 (1.11)		3.37 (1.05)		3.21 (1.12)		3.18 (1.08)		3.02 (1.18)	
Parents who smoke tobacco, no. (%)										
None	297 (36.5)		73 (35.6)		70 (34.3)		75 (36.9)		79 (39.3)	
One	266 (32.7)		59 (28.8)		64 (31.4)		66 (32.5)		77 (38.3)	
Both	250 (30.8)		73 (35.6)		70 (34.3)		62 (30.5)		45 (22.4)	

Mean (SD) unless otherwise noted.

Abbreviations: SD = standard deviation.

Supplementary Table 3: Adjusted associations of early-life growth conditions with adult height-GaP in ALSPAC.

	**Adjusted mean difference in height-GaP, cm (95%CI) p-value**
Pregnancy index of multiple deprivation, per 1-quintile increment	-0.16 (-0.28, -0.05) p=0.006
Maternal smoking during pregnancy	p=0.001
Never	Reference
1-4 cigarettes/day, n (%)	-1.03 (-1.86, -0.20)
5-9 cigarettes/day, n (%)	-1.05 (-1.93, -0.17)
10-19 cigarettes/day, n (%)	-0.61 (-1.31, 0.09)
20+ cigarettes/day, n (%)	-1.51 (-2.71, -0.30)
"Healthy" diet principal component during pregnancy, per 1-SD decrement	-0.43 (-0.59, -0.27) p<0.001
Gestational age at birth, weeks, no. (%)	p=0.005
38+	Reference
32-<38	-0.08 (-0.59, 0.43)
<32	-4.09 (-7.14, -1.03)
Birth length, per 1-cm decrement	-0.67 (-0.78, -0.55) p<0.001
Birth weight, per 1-kg decrement	-2.22 (-2.63, -1.82) p<0.001
Breastfeeding duration	p<0.001
6+ months	Reference
3-<6 months	-0.06 (-0.53, 0.42)
1-<3 months	-0.56 (-1.04, -0.08)
<1 month	-0.55 (-1.05, -0.05)
Never	-1.03 (-1.50, -0.56)
Childhood (ages 0-12 years) index of multiple deprivation, per 1-quintile increment	-0.22 (-0.34, -0.09) p=0.001
Childhood (ages 6-34 months) household tobacco smoke exposure duration	p<0.001
0 hours/week	Reference
>0-5 hours/week	-0.34 (-0.70, 0.01)
>5-10 hours/week	-0.99 (-1.66, -0.31)
>10-20 hours/week	-1.22 (-1.86, -0.57)
20+ hours/week	-1.03 (-1.64, -0.42)
"Healthy" diet principal component at 38 months, per 1-SD decrement	-0.22 (-0.41, -0.03) p=0.026
Residential outdoor $PM_{2.5}$ concentration, per 1-μg/m^3 increment	0.06 (-0.15,0.26) p=0.593

Model covariables: age at height-GaP assessment, sex, genotype-predicted height. P-values reflect two-sided Wald tests. The p-value threshold to infer statistical significance was set at 0.050/5=0.010 to account for testing of five early-life growth factors (nutrition, peri-natal, noxious exposures, and socio-economic deprivation). Abbreviations: ALSPAC = Avon Longitudinal Study of Parents and Children; SD = standard deviation; $PM_{2.5}$ = particulate matter with diameter less the 2.5 micrometres.

Supplementary Table 4: Unadjusted associations of early-life growth conditions with adult height-GaP in ALSPAC.

	**Unadjusted mean difference in height-GaP, cm (95%CI)**
Pregnancy index of multiple deprivation, per 1-quintile increment	-0.19 (-0.31, -0.08) p=0.001
Maternal smoking during pregnancy	p=0.001
Never	Reference
1-4 cigarettes/day, n (%)	-1.06 (-1.90, -0.23)
5-9 cigarettes/day, n (%)	-1.09 (-1.98, -0.21)
10-19 cigarettes/day, n (%)	-0.64 (-1.34, 0.06)
20+ cigarettes/day, n (%)	-1.54 (-2.75, -0.33)
"Healthy" diet principal component during pregnancy, per 1-SD decrement	-0.46 (-0.62, -0.30) p<0.001
Gestational age at birth, weeks, no. (%)	p=0.005
38+	Reference
32-<38	-0.07 (-0.58, 0.44)
<32	-4.14 (-7.22, -1.05)
Birth length, per 1-cm decrement	-0.62 (-0.72, -0.51) p<0.001
Birth weight, per 1-kg decrement	-2.16 (-2.55, -1.77) p<0.001
Breastfeeding duration	p<0.001
6+ months	Reference
3-<6 months	-0.06 (-0.54, 0.42)
1-<3 months	-0.60 (-1.08, -0.12)
<1 month	-0.62 (-1.12, -0.11)
Never	-1.12 (-1.59, -0.64)
Childhood (ages 0-12 years) index of multiple deprivation, per 1-quintile increment	-0.25 (-0.38, -0.13) p<0.001
Childhood (ages 6-34 months) household tobacco smoke exposure duration	p<0.001
0 hours/week	Reference
>0-5 hours/week	-0.40 (-0.75, -0.04)
>5-10 hours/week	-1.08 (-1.76, -0.40)
>10-20 hours/week	-1.31 (-1.96, -0.67)
20+ hours/week	-1.13 (-1.74, -0.51)
"Healthy" diet principal component at 38 months, per 1-SD decrement	-0.21 (-0.40, -0.01)
Residential outdoor $PM_{2.5}$ concentration, per 1-μg/m^3 increment	0.06 (-0.15, 0.26) p=0.595

P-values reflect two-sided Wald tests. The p-value threshold to infer statistical significance was set at 0.050/5=0.010 to account for testing of five early-life growth factors (nutrition, peri-natal, noxious exposures, and socio-economic deprivation). Abbreviations: ALSPAC = Avon Longitudinal Study of Parents and Children; SD = standard deviation; $PM_{2.5}$ = particulate matter with diameter less the 2.5 micrometres.

Supplementary Table 5: Adjusted associations of early-life growth conditions with adult height-GaP in the Dunedin Multidisciplinary Health and Development Study.

	**Adjusted mean difference in height-GaP cm (95%CI) p-value**
Maternal smoking during pregnancy	p=0.239
No	Reference
Yes	-0.46 (-1.22, 0.30)
Gestational age at birth, weeks, no. (%)	p=0.347
38+	Reference
32-<38	0.47 (-0.82, 1.75)
<32	-6.62 (-17.01, 3.78)
Birth length, cm, per 1-cm decrement	-0.63 (-0.78, -0.47) p<0.001
Birth weight, kg, per 1-kg decrement	-2.43 (-3.11, 1.75) p<0.001
Breastfeeding duration	p=0.001
3+ months	Reference
0-<3 months	-0.96 (-1.90, -0.01)
Never	-1.64 (-2.51, -0.77)
Childhood (ages 0-15 years) index of socio-economic deprivation, per 1-SD increment	-0.63 (-0.98, -0.28) p=0.001
Tobacco-smoking parents	p=0.004
None	Reference
One	-0.96 (-1.90, -0.01)
Both	-1.64 (-2.51, -0.77)

Model covariables: age at height-GaP assessment, sex, genotype-predicted height. P-values reflect two-sided Wald tests and are not adjusted for multiple testing. The p-value threshold to infer statistical significance for this replication analysis was set at 0.050. Abbreviations: SD = standard deviation.

Supplementary Table 6: Association of measured height, genotype-predicted height and height-GaP with mortality in UKBiobank and MESA.

	**UKBiobank**			**MESA**		
	**Hazard ratio per 1-SD deficit in Measured height**	**Hazard ratio per 1-SD deficit in genotype-predicted height**	**Hazard ratio per 1-SD deficit in Height-GaP**	**Hazard ratio per 1-SD deficit in measured height**	**Hazard ratio per 1-SD deficit in genotype-predicted height**	**Hazard ratio per 1-SD deficit in height-GaP**
All-cause death	1.08 (1.06, 1.09) p<0.001	0.98 (0.97, 0.99) p<0.001	1.11 (1.10, 1.12) p<0.001	1.08 (1.01, 1.15) p=0.016	1.00 (0.93, 1.08) p=0.971	1.09 (1.03, 1.15) p=0.004
Atherosclerotic cardiovascular disease death	1.12 (1.10, 1.15) p<0.001	1.01 (0.98, 1.03) p=0.513	1.15 (1.13, 1.18) p<0.001	1.14 (0.98, 1.32) p=0.095	0.97 (0.82, 1.15) p=0.740	1.17 (1.02, 1.34) p=0.027
Atherosclerotic coronary heart disease death	1.22 (1.18, 1.26) p<0.001	1.03 (1.00, 1.07) p=0.064	1.24 (1.21, 1.28) p<0.001	1.31 (1.09, 1.57) p=0.005	0.98 (0.79, 1.21) p=0.846	1.35 (1.14, 1.59) p<0.001

The models estimating the measured height associations with mortality included age, sex, principal components of genetic ancestry and measured height.
The models estimating the genotype-predicted height and height-GaP associations with mortality included age, sex and principal components of genetic ancestry, genotype-predicted height and height-GaP. P-values reported from two-sided Wald tests. The p-value threshold to infer statistical significance was set at 0.050/3=0.017 to account for testing three mortality outcomes in UKBiobank and p-value=0.050 for replication in MESA. Abbreviations: MESA = Multi-Ethnic Study of Atherosclerosis, SD = standard deviation.

Supplementary Table 7: Association of height-GaP with mortality in UKBiobank accounting for later-life height loss.

	**Hazard ratio per 1-SD height-GaP deficit (95%CI) p-value**	
	**Median height-loss corrected height-GaP**	**Worst case scenario height-loss corrected height-GaP**
All-cause death		
Model 1	1.11 (1.10, 1.12) p<0.001	1.07 (1.06, 1.07) p<0.001
Model 2	1.11 (1.10, 1.13) p<0.001	1.07 (1.06, 1.07) p<0.001
Atherosclerotic cardiovascular disease death		
Model 1	1.14 (1.12, 1.17) p<0.001	1.09 (1.07, 1.10) p<0.001
Model 2	1.14 (1.12, 1.17) p<0.001	1.09 (1.07, 1.10) p<0.001
Atherosclerotic coronary heart disease death		
Model 1	1.24 (1.20, 1.27) p<0.001	1.14 (1.12, 1.17) p<0.001
Model 2	1.24 (1.20, 1.27) p<0.001	1.14 (1.12, 1.17) p<0.001

Model 1: Age, sex, principal components of genetic ancestry. Model 2: Model 1 variables, genotype-predicted height. P-values reported from two-sided Wald tests. The p-value threshold to infer statistical significance was set at 0.050/3=0.017 to account for testing three mortality outcomes in UKBiobank. Abbreviations: SD = standard deviation.

Supplementary Table 8: Association of height-GaP with mortality adjusted for adult health-related factors in UKBiobank and MESA.

	**Hazard ratio per 1-SD height-GaP deficit (95%CI) p-value**	
	**UKBiobank**	**MESA**
All-cause death	1.04 (1.03, 1.05) p<0.001	1.09 (1.03, 1.16) p=0.006
Atherosclerotic cardiovascular disease death	1.05 (1.03, 1.08) p<0.001	1.18 (1.02, 1.36) p=0.022
Atherosclerotic coronary heart disease death	1.12 (1.08, 1.16) p<0.001	1.32 (1.10, 1.57) p=0.003

Model covariables: age, sex, principal components of genetic ancestry, genotype-predicted height, cigarette smoking status, pack-years, alcohol use status, drinks per week, minutes of moderate and of vigorous physical activity per week, weight class, diabetes status, hypertension status, systolic blood pressure, LDL cholesterol concentration, cholesterol-lowering medication use, educational attainment, health insurance status (in MESA) and household income. P-values reported from two-sided Wald tests. The p-value threshold to infer statistical significance was set at 0.050/3=0.017 to account for testing three mortality outcomes in UKBiobank and p-value=0.050 for replication in MESA. Abbreviations: MESA = Multi-Ethnic Study of Atherosclerosis, SD = standard deviation.

Supplementary Table 9: Association of early-life growth conditions with adult height-GaP in ALSPAC restricted to participants with non-missing data.

	**Adjusted mean difference in height-GaP (95%CI) p-value**
Pregnancy index of multiple deprivation, per 1-quintile increment	-0.19 (-0.30, -0.08) p=0.001
Maternal smoking during pregnancy	p<0.001
Never	Reference
1-4 cigarettes/day, n (%)	-1.07 (-1.90, -0.23)
5-9 cigarettes/day, n (%)	-1.07 (-1.96, -0.18)
10-19 cigarettes/day, n (%)	-0.68 (-1.38, 0.02)
20+ cigarettes/day, n (%)	-1.71 (-2.92, -0.50)
"Healthy" diet principal component during pregnancy, per 1-SD decrement	-0.43 (-0.59, -0.27) p<0.001
Gestational age at birth, weeks, no. (%)	p<0.001
38+	Reference
32-<38	-0.07 (-0.58, 0.43)
<32	-5.37 (-7.23, -3.50)
Birth length, cm, per 1-SD decrement	-0.73 (-0.82, -0.65) p<0.001
Birth weight, kg, per 1-SD decrement	-2.41 (-2.68, -2.13) p<0.001
Breastfeeding duration	p<0.001
6+ months	Reference
3-<6 months	-0.07 (-0.54, 0.40)
1-<3 months	-0.60 (-1.06, -0.14)
<1 month	-0.56 (-1.04, -0.09)
Never	-1.07 (-1.53, -0.62)
Childhood (ages 0-12 years) index of multiple deprivation, per 1-quintile increment	-0.23 (-0.36, -0.11) p<0.001
Childhood (ages 6-34 months) household tobacco smoke exposure duration	p<0.001
0 hours/week	Reference
>0-5 hours/week	-0.28 (-0.67, 0.11)
>5-10 hours/week	-1.50 (-2.27, -0.73)
>10-20 hours/week	-1.30 (-2.05, -0.55)
20+ hours/week	-0.95 (-1.71, -0.19)
"Healthy" diet principal component at 38 months, per 1-SD decrement	-0.23 (-0.39, -0.07) p=0.005
Residential outdoor $PM_{2.5}$ concentration, μg/m^3, per 1-SD decrement	0.04 (-0.16, 0.24) p=0.704

Model covariables: age at height-GaP assessment, sex and genotype-predicted height. P-values reflect two-sided Wald tests. The p-value threshold to infer statistical significance was set at 0.050/5=0.010 to account for testing of five early-life growth factors (nutrition, peri-natal, noxious exposures, and socio-economic deprivation). Abbreviations: ALSPAC = Avon Longitudinal Study of Parents and Children; SD = standard deviation; $PM_{2.5}$ = particulate matter with diameter less the 2.5 micrometres.

Supplementary Table 10: Association of early-life growth conditions with adult height-GaP in ALSPAC restricted to participants with height-GaP assessed at the age 24 study visit.

	**Adjusted mean difference in height-GaP (95%CI) p-value**
Pregnancy index of multiple deprivation, per 1-quintile increment	-0.13 (-0.27, 0.08) p=0.069
Maternal smoking during pregnancy	p=0.014
Never	Reference
1-4 cigarettes/day, n (%)	-0.74 (-1.77, 0.28)
5-9 cigarettes/day, n (%)	-1.20 (-2.29, -0.11)
10-19 cigarettes/day, n (%)	-0.91 (-1.77, -0.04)
20+ cigarettes/day, n (%)	-1.34 (-2.87, 0.20)
"Healthy" diet principal component during pregnancy, per 1-SD decrement	-0.50 (-0.70,-0.30) p<0.001
Gestational age at birth, weeks, no. (%)	P=0.009
38+	Reference
32-<38	-0.25 (-0.87, 0.37)
<32	-5.93 (-10.86, -0.99)
Birth length, cm, per 1-SD decrement	-0.68 (-0.82, -0.54) p<0.001
Birth weight, kg, per 1-SD decrement	-2.34 (-2.83,-1.85) p<0.001
Breastfeeding duration	P=0.014
6+ months	Reference
3-<6 months	-0.11 (-0.66, 0.45
1-<3 months	-0.43 (-0.99, 0.13)
<1 month	-0.41 (-1.00, 0.19)
Never	-0.96 (-1.52, -0.39)
Childhood (ages 0-12 years) index of multiple deprivation, per 1-quintile increment	-0.13 (-0.26, 0.01) p=0.063
Childhood (ages 6-34 months) household tobacco smoke exposure duration	p<0.001
0 hours/week	Reference
>0-5 hours/week	-0.39 (-0.80, 0.03)
>5-10 hours/week	-1.27 (-2.12, -0.41)
>10-20 hours/week	-1.26 (-2.07, -0.46)
20+ hours/week	-1.20 (-1.98, -0.42)
"Healthy" diet principal component at 38 months, per 1-SD decrement	-0.19 (-0.40, 0.03,) p=0.086
Residential outdoor $PM_{2.5}$ concentration, $\mu g/m^3$, per 1-SD decrement	0.10 (-0.14, 0.34) p=0.421

Model covariables: age at height-GaP assessment, sex and genotype-predicted height. P-values reflect two-sided Wald tests. The p-value threshold to infer statistical significance was set at 0.050/5=0.010 to account for testing of five early-life growth factors (nutrition, peri-natal, noxious exposures, and socio-economic deprivation). Abbreviations: ALSPAC = Avon Longitudinal Study of Parents and Children; SD = standard deviation; $PM_{2.5}$ = particulate matter with diameter less the 2.5 micrometres.

Supplementary Table 11: Association of ancestry-specific polygenic height score-derived height-GaP with mortality in UKBiobank and MESA.

	**Hazard ratio per 1-SD height-GaP deficit (95%CI) p-value**	
	**UKBiobank**	**MESA**
All-cause death		
Model 1	1.12 (1.10, 1.13) p<0.001	1.08 (1.02, 1.14) p=0.012
Model 2	1.12 (1.10, 1.13) p<0.001	1.08 (1.02, 1.14) p=0.011
Atherosclerotic cardiovascular disease death		
Model 1	1.15 (1.13, 1.18) p<0.001	1.13 (1.00, 1.27) p=0.046
Model 2	1.15 (1.13, 1.18) p<0.001	1.12 (1.00, 1.26) p=0.049
Atherosclerotic coronary heart disease death		
Model 1	1.25 (1.21, 1.29) p<0.001	1.27 (1.07, 1.51) p=0.005
Model 2	1.25 (1.21, 1.29) p<0.001	1.28 (1.08, 1.52) p=0.004

Model 1: age, sex, principal components of genetic ancestry. Model 2: Model 1 variables, genotype-predicted height. P-values reported from two-sided Wald tests. The p-value threshold to infer statistical significance was set at 0.050/3=0.017 to account for testing three mortality outcomes in UKBiobank and p-value=0.050 for replication in MESA. Abbreviations: MESA = Multi-Ethnic Study of Atherosclerosis; SD = standard deviation.

Supplementary Table 12: Association of height-GaP with mortality in UKBiobank and MESA using genotype-predicted height derived from cohort- and sex-specific regression models of measured height.

	**Hazard ratio per 1-SD height-GaP deficit (95%CI) p-value**	
	**UKBiobank**	**MESA**
All-cause death		
Model 1	1.11 (1.10, 1.13) p<0.001	1.10 (1.03, 1.17) p=0.005
Model 2	1.12 (1.10, 1.13) p<0.001	1.09 (1.01, 1.17) p=0.019
Atherosclerotic cardiovascular disease death		
Model 1	1.15 (1.13, 1.18) p<0.001	1.15 (0.99, 1.35) p=0.075
Model 2	1.15 (1.13, 1.18) p<0.001	1.19 (1.01, 1.40) p=0.038
Atherosclerotic coronary heart disease death		
Model 1	1.25 (1.22, 1.29) p<0.001	1.38 (1.12, 1.69) p=0.002
Model 2	1.25 (1.22, 1.29) p<0.001	1.35 (1.12, 1.64) p=0.002

Model 1: age, sex, principal components of genetic ancestry. Model 2: Model 1 variables, genotype-predicted height. P-values reported from two-sided Wald tests. The p-value threshold to infer statistical significance was set at 0.050/3=0.017 to account for testing three mortality outcomes in UKBiobank and p-value=0.050 for replication in MESA. Abbreviations: MESA = Multi-Ethnic Study of Atherosclerosis; SD = standard deviation.
