# Supplementary material for: Quantifying the impact of early life growth adversity on later life health
Source: Commun Med (Lond). 2025 Nov 17;5:534. doi: 10.1038/s43856-025-01245-3 (PMC12749450; doi:10.1038/s43856-025-01245-3)
Supplement: Supplementary file 3 — Description of Additional Supplementary Files [file 43856_2025_1245_MOESM3_ESM.docx]

**Description of Additional Supplementary Files**

Supplementary Data 1: Definitions and units of variables included in this study by cohort:

Supplementary Data 2: Characteristics of participants included in the ALSPAC analyses

Supplementary Data 3: Characteristics of participants included in the UKBiobank analyses.

Supplementary Data 4: Characteristics of participants in the UKBiobank included and excluded from the analysis

Supplementary Data 5: Characteristics of participants included in the Multi-Ethnic Study of Atherosclerosis analyses.

Supplementary Data 6: Source Data
