# Supplementary material for: Quantifying the impact of early life growth adversity on later life health
Source: Commun Med (Lond). 2025 Nov 17;5:534. doi: 10.1038/s43856-025-01245-3 (PMC12749450; doi:10.1038/s43856-025-01245-3)
Supplement: Supplementary file 4 — Supplementary Data 1 [file 43856_2025_1245_MOESM4_ESM.docx]

*Supplementary Data 1:* Definitions and units of variables included in this study by cohort:

| **Variable** | **Cohort** | | | |
| --- | --- | --- | --- | --- |
|  | **ALSPAC** | **DMHDS** | **UKBiobank** | **MESA** |
| Age | Date of birth to study visit, years (integer) | Date of birth to study visit, years (integer) | Date of birth to study visit, years (integer) | Self-reported date of birth to study visit, years (integer) |
| Sex | Self-report, fixed category | Documented at birth, fixed category | Self-report, fixed category | Self-report, fixed category: male, female |
| Height | Standing shoeless, cm, integer | Standing shoeless, cm, integer | Standing shoeless, cm, integer | Standing shoeless, cm, integer |
| Adult weight | NA | NA | Standing bare foot on a Tanita BC418MA body composition analyzer | Standing shoeless, in light clothes with pockets emptied and jewelry removed, kg |
| Adult body mass index class | NA | NA | BMI category labelling based on continuous measurements:  <18.5 kg/m^2^  18.5 to <25 kg/m^2^  25 to <30 kg/m^2^  30+ kg/m^2^ | <18.5 kg/m^2^  18.5 to <25 kg/m^2^  25 to <30 kg/m^2^  30+ kg/m^2^ |
| Gestational age at birth | Time from date of mother’s last menstrual period to birth, weeks, based on mother’s self-report or clinical ultrasound assessment | Calculated from the date of last menstrual period when this was recalled with confidence. | NA | NA |
| Birth weight | Derived from birthweight data from obstetric data, ALSPAC measures and from birth notification/obstetric data, kilograms:  1. Value taken if identical from all sources  2. Lower value taken if disagreement between data sources <100g | Measured at birth, kilograms | NA | NA |
| Birth length | Crown-heel length using ALSPAC measures, cm | Measured at birth, mm. | NA | NA |
| Breastfeeding status and duration | Child-based questionnaire at 6 months of age, duration of breast feeding, fixed category:  1. <1 month  2. 1-<3 months  3. 3-<6 months  4. 6 or more  5. Missing | Number of weeks of breastfeeding reported by mother at the age 3 years assessment and validated from visiting nurse records | NA | NA |
| Index of multiple deprivation during pregnancy | Deprivation index in quintiles:  1 = low deprivated  2 = medium-low deprivated  3 = medium deprivated  4 = medium-high deprivated  5 = high deprivated | NA | NA | NA |
| Index of multiple deprivation during childhood | Mean deprivation index from birth to age 12 years. Higher quintiles indicate higher levels of deprivation. | Socioeconomic status based on the highest parental occupation recorded at each assessment between birth and age 15 years. Occupations were graded from 1 (high) to 6 (low) based on the income and education associated with that occupation in the New Zealand census. | NA | NA |
| Maternal smoking during pregnancy | No. of times smoked per day in the last two weeks, mother-based questionnaire at 18w gestation, category:  1. 0  2. 1-4  3. 5-9  4. 10-19  6. 20+  7. Missing | NA | NA | NA |
| Maternal “healthy” diet principal component during pregnancy | Derived dietary pattern scores for mother at 32 weeks pregnancy, component ‘Healthy’, according to mother-based questionnaire at 38 weeks gestation | NA | NA | NA |
| Household tobacco smoke during childhood | Estimated second-hand exposure to tobacco smoke, hours/week, from ages 6 months to 4.5 years. Categories: 1. 0  2. 1-4  4. 5-9  5. 10-19  6. 20+  7. Missing | Either parent reported to smoke at the age 7, 9, 11, or 13 year-old assessments. | NA | NA |
| “Healthy” diet principal component at 38 months | Derived dietary pattern scores at 38m age, component ‘Healthy’, according to child-based questionnaire at 3y2m of age | NA | NA | NA |
| Residential outdoor PM_2.5_ concentration | Particulate Matter <2.5 μg exposure average value at pregnancy, back-extrapolated using ratio method, μg/m^3^ | NA | NA | NA |
| Educational attainment | NA | NA | Self-reported, fixed category, multi-selection:  1. College or University degree  2. A levels/AS levels or equivalent  3. O levels/GCSEs or equivalent  4. CSEs or equivalent  5. NVQ or HND or HNC or equivalent  6. Other professional qualifications eg: nursing, teaching  7. None of the above  8. Prefer not to answer | Self-reported, fixed category:  1. No schooling  2. Grades 1-8  3. Grades 9-11  4. Completed high school or general educational development  5. Some college but no degree  6. Technical school certificate  7. Associate degrees  8. Bachelor’s degree  9. Graduate or professional degree |
| Family income, past 12 months | NA | NA | Self-reported, pre-tax, fixed category (£Pounds):  1. Less than 18,000  2. 18,000 to 30,999  3. 31,000 to 51,999  4. 52,000 to 100,000  5. Greater than 100,000  6. Do not know  7. Prefer not to answer | Self-reported, fixed category ($USD):  1: < $5,000  2: $5,000-7,999  3: $8,000-11,999  4: $12,000-15,999  5: $16,000-19,999  6: $20,000-24,999  7: $25,000-29,999  8: $30,000-34,999  9: $35,000-39,999  10: $40,000-49,999  11: $50,000-74,999  12: $75,000-99,999  13: $100,000+ |
| Health insurance status | NA | NA | NA | Self-reported, fixed categories:  1. Private  2. Medicare  3. Medicare + private  4. Medicaid  5. Military / Veteran affairs  6. Other  7. None |
| Race-ethnicity | Self-reported, fixed categories | Self-reported, fixed categories | Self-reported, fixed categories | Self-reported, fixed categories:  1. White  2. Black  3. Hispanic/Latino  4. Chinese |
| Cigarette smoking status | NA | NA | Self-reported | Self-reported |
| Never | NA | NA | Self-reported absence of past or former smoking behaviour | <100 lifetime cigarettes smoked |
| Former | NA | NA | Self-reported past tobacco smoking ‘smoked occasionally’ or ‘Smoked on most or all days’ | >=100 lifetime cigarettes smoked and cigarette smoked >30 days ago |
| Current | NA | NA | Self-reported current smoking ‘on most or all days’ or ‘Only occasionally’ | Cigarette smoked within 30 days |
| Pack-years | NA | NA | Number of cigarettes smoked per day / 20 * number of years smoking  Individuals who gave up smoking for >6 months is adjusted: Number of cigarettes smoked per day / 20 * (number of years smoking – 0.5) | Average number of cigarettes smoked per day while smoking / 20 * number of years smoking |
| Alcohol drinking status | NA | NA | Self-reported never, previous, current | Self-reported never, former, current |
| Drinks per week | NA | NA | Self-reported, fixed categories:  1. Daily or almost daily  2. Three or four times a week  3. Once or twice a week  4. One to three times a month  5. Special occasions only  6. Never  7. Prefer not to answer | Average number of drinks per week when drinking |
| Diabetes status | NA | NA | Self-reported previous diagnosis, yes, no, do not know, prefer not to answer | Fasting glucose >=126 mg/dl or use of glucose lowering medications |
| Hypertension status | NA | NA | Systolic blood pressure >= 140 mmHg or diastolic blood pressure > 90 mmHg or previous diagnosis of cardiovascular disease | Systolic blood pressure >= 140 mmHg or diastolic blood pressure >= 90 mmHg or hypertension medication use |
| Systolic blood pressure | NA | NA | Automated reading, mmHg | Average of 2nd and 3rd Dinamap systolic blood pressure measurements, in mm Hg |
| Diastolic blood pressure | NA | NA | Automated reading, mmHg | Average of 2nd and 3rd Dinamap diastolic blood pressure measurements, in mm Hg |
| Low density lipoprotein cholesterol concentration | NA | NA | Measured from randomly selected EDTA plasma samples using high-throughput NMR-based metabolic profiling platform, mmol/l, continuous | Friedewald equation-derived low density lipoprotein cholesterol concentration from overnight fasting serum measured total cholesterol, high density lipoprotein cholesterol and triglyceride |
| Hypertension medication use | NA | NA | Self-reported | Medication inventory |
| Diabetes medication use | NA | NA | Self-reported | Medication inventory |
| Lipid lowering medication use | NA | NA | Self-reported | Medication inventory |
| Moderate physical activity | NA | NA | Derived according to IPAQ guidelines, minutes/week, continuous | Self-reported questionnaire (Typical Week Physical Activity Survey)-derived moderate physical activity metabolic equivalent-minutes per week |
| Vigorous physical activity | NA | NA | Derived according to IPAQ guidelines, minutes/week, continuous | Self-reported questionnaire (Typical Week Physical Activity Survey)-derived vigorous physical activity metabolic equivalent-minutes per week |
| Death status | NA | NA | Derived from primary cause of death (ICD 10) according to National Death Registries | Derived from interval household phone contact every 9-12 months and query to National Death Index. |
| Death attributed to atherosclerotic cardiovascular disease | NA | NA | Derived from primary cause of death (ICD 10) according to National Death Registries, categories include:  I20-25 Ischemic heart diseases  I60 Subarachnoid haemorrhage  I61 Intracerebral haemorrhage  I63 Cerebral infarction  I64 Stroke, not specified as haemorrhage or infarction | Determined via standardized adjudication that included paired cardiologist or neurologist review of abstracted medical records, with disagreements resolved by full committee review. |
| Time to death | NA | NA | Date of death – date of enrollment divided by 365.25 days | Date of death – date of enrollment divided by 365.25 days |

Data dictionaries can be accessed at: <http://www.bristol.ac.uk/alspac/researchers/our-data/> <https://biobank.ndph.ox.ac.uk/showcase/> <https://dunedinstudy.otago.ac.nz/> <https://www.mesa-nhlbi.org/>

Abbreviations: ALSPAC = Avon Longitudinal Study of Parents and Children; DMHDS = Dunedin Multi-disciplinary Health and Development Study; MESA = Multi-Ethnic Study of Atherosclerosis; PM_2.5_ = particulate matter with diameter less than 2.5 micrometres; NA = not applicable
