# Supplementary material for: Quantifying the impact of early life growth adversity on later life health
Source: Commun Med (Lond). 2025 Nov 17;5:534. doi: 10.1038/s43856-025-01245-3 (PMC12749450; doi:10.1038/s43856-025-01245-3)
Supplement: Supplementary file 5 — Supplementary Data 2 [file 43856_2025_1245_MOESM5_ESM.docx]

*Supplementary Data 2:* Characteristics of participants included in the ALSPAC analyses.

|  | **All** | | **By Height-GaP Quartile** | | | | | | | | **P-Value** | |
| --- | --- | --- | --- | --- | --- | --- | --- | --- | --- | --- | --- | --- |
|  |  |  | **1** | | **2** | | **3** | | **4** | |  | |
| No. | 4,582 | | 1,146 | | 1,146 | | 1,145 | | 1,145 | |  | |
| Sex, no. (%) |  | |  | |  | |  | |  | | 0.763 | |
| Female | 2,579 (56.3) | | 643 (56.1) | | 656 (57.2) | | 649 (56.7) | | 631 (55.1) | |  | |
| Male | 2,003 (43.7) | | 503 (43.9) | | 490 (42.8) | | 496 (43.3) | | 514 (44.9) | |  | |
| Age at height-GaP assessment, years, median (IQR) | 24 (18, 25) | | 24 (18, 25) | | 24 (18, 25) | | 24 (19, 25) | | 24 (18, 25) | | 2.85  ×10^-8^ | |
|  | Male | Female | Male | Female | Male | Female | Male | Female | Male | Female |  | |
| Genotype-predicted height, cm | 179.6  (4.3) | 165.9  (3.9) | 179.7  (4.2) | 165.9 (3.8) | 179.4  (4.3) | 165.8  (3.8) | 179.5  (4.3) | 165.7  (3.9) | 179.6  (4.4) | 166.0  (4.0) | 0.385 |  |
| Height-GaP, cm | 0.00  (5.24) | 0.00  (4.9) | -6.6  (2.9) | -6.2 (2.5) | -1.7  (0.9) | -1.5  (1.0) | 1.6  (0.9) | 1.6  (0.9) | 6.5  (2.6) | 6.3  (2.7) | <1.00×10^-100^ |  |
| Measured height, cm | 179.6  (6.8) | 165.9  (6.3) | 173.1  (5.0) | 159.7 (4.4) | 177.8  (4.4) | 164.2  (3.8) | 181.1  (4.4) | 167.3  (4.0) | 186.1  (5.3) | 172.3  (4.9) | <1.00×10^-100^ | |
| Pregnancy index of multiple deprivation quintile, mean | 2.91 (1.38) | | 3.04 (1.40) | | 2.94 (1.36) | | 2.91 (1.37) | | 2.78 (1.36) | | 4.45  ×10^-4^ | |
| Maternal smoking during pregnancy, no. (%) |  | |  | |  | |  | |  | | 0.003 | |
| Never | 3,751 (87.3) | | 988 (83.6) | | 931 (87.1) | | 944 (87.9) | | 977 (90.5) | |  | |
| 1-4 cigarettes/day, n (%) | 144 (3.4) | | 46 (4.3) | | 41 (3.8) | | 31 (2.9) | | 26 (2.4) | |  | |
| 5-9 cigarettes/day, n (%) | 126 (2.9) | | 39 (3.6) | | 33 (3.1) | | 34 (3.2) | | 20 (1.9) | |  | |
| 10-19 cigarettes/day, n (%) | 209 (4.9) | | 63 (5.9) | | 51 (4,8) | | 51 (4.7) | | 44 (4.1) | |  | |
| 20+ cigarettes/day, n (%) | 67 (1.6) | | 28 (2.6) | | 13 (1.2) | | 14 (1.3) | | 12 (1.1) | |  | |
| “Healthy” diet principal component during pregnancy | 0.24 (0.95) | | 0.14 (0.96) | | 0.24 (0.95) | | 0.26 (0.95) | | 0.34 (0.92) | | 1.53  ×10^-5^ | |
| Gestational age at birth, weeks | 39.5 (1.8) | | 39.3 (2.0) | | 39.5 (1.8) | | 39.5 (1.8) | | 39.5 (1.7) | | 0.095 | |
| Birth length, cm | 50.6 (2.1) | | 49.9 (2.1) | | 50.4 (2.1) | | 50.8 (2.0) | | 51.4 (2.0) | | 5.87  ×10^-41^ | |
| Birth weight, kg | 3.43 (0.53) | | 3.26 (0.57) | | 3.40 (0.52) | | 3.47 (0.50) | | 3.57 (0.50) | | 1.60  ×10^-43^ | |
| Breastfeeding duration, no. (%) |  | |  | |  | |  | |  | | 0.004 | |
| Never | 641 (15.8) | | 192 (19.1) | | 166 (16.5) | | 158 (15.6) | | 125 (12.1) | |  | |
| <1 month | 580 (14.3) | | 158 (15.7) | | 136 (13.5) | | 143 (14.1) | | 143 (13.9) | |  | |
| 1-<3 months | 623 (15.4) | | 159 (15.8) | | 157 (15.6) | | 159 (15.7) | | 148 (14.4) | |  | |
| 3-<6 months | 601 (14.8) | | 138 (13.7) | | 145 (14.4) | | 151 (14.9) | | 167 (16.2) | |  | |
| 6+ months | 1608 (39.7) | | 358 (35.6) | | 401 (39.9) | | 401 (39.6) | | 448 (43.5) | |  | |
| Childhood index of multiple deprivation quintile, mean | 2.77 (1.26) | | 2.89 (1.29) | | 2.79 (1.24) | | 2.77 (1.27) | | 2.64 (1.24) | | 3.37  ×10^-4^ | |
| Childhood household tobacco smoke exposure duration, no. (%) |  | |  | |  | |  | |  | | 0.002 | |
| 0 hours/week | 1,813 (54.2) | | 388 (48.2) | | 446 (54.6) | | 476 (55.4) | | 503 (58.2) | |  | |
| >0-5 hours/week | 984 (29.4) | | 241 (29.9) | | 238 (29.1) | | 254 (29.6) | | 251 (29.0) | |  | |
| >5-10 hours/week | 177 (5.3) | | 56 (7.0) | | 47 (5.8) | | 41 (4.8) | | 33 (3.8) | |  | |
| >10-20 hours/week | 189 (5.6) | | 61 (7.6) | | 45 (5.5) | | 45 (5.2) | | 38 (4.4) | |  | |
| 20+ hours/week | 183 (5.5) | | 59 (7.3) | | 41 (5.0) | | 43 (5.0) | | 40 (4.6) | |  | |
| “Healthy” diet principal component at 38 months | 0.05 (0.99) | | -0.01 (0.91) | | 0.07 (0.99) | | 0.06 (1.00) | | 0.08 (1.03) | | 0.156 | |
| Residential outdoor PM_2.5_ concentration, μg/m^3^ | 13.26 (0.77) | | 13.24 (0.74) | | 13.28 (0.75) | | 13.27 (0.79) | | 13.25 (0.79) | | 0.703 | |

Mean (SD) unless otherwise specified. The thresholds to define height-GaP quartile membership were computed for each sex; small imbalances in the number of participants per quartile reflect the recording of participant height to the nearest centimeter. P-values reflect two-sided chi-squared, Kruskal-Wallis, or Welch tests and were not corrected for multiple testing.

Abbreviations: ALSPAC = Avon Longitudinal Study of Parents and Children; PM_2.5_ = particulate matter <2.5 micrometres in diameter.
