# Supplementary material for: Quantifying the impact of early life growth adversity on later life health
Source: Commun Med (Lond). 2025 Nov 17;5:534. doi: 10.1038/s43856-025-01245-3 (PMC12749450; doi:10.1038/s43856-025-01245-3)
Supplement: Supplementary file 6 — Supplementary Data 3 [file 43856_2025_1245_MOESM6_ESM.docx]

*Supplementary Data 3:* Characteristics of participants included in the UKBiobank analyses*.*

|  | **All** | | **By Height-GaP Quartile** | | | | | | | |  |
| --- | --- | --- | --- | --- | --- | --- | --- | --- | --- | --- | --- |
|  |  |  | **1** | | **2** | | **3** | | **4** | | **P-value** |
| No. | 483,385 | | 120,847 | | 120,846 | | 120,845 | | 120,847 | |  |
| Sex, no. (%) |  | |  | |  | |  | |  | | 1.000 |
| Female | 262,338 (54.3) | | 65,585 (54.3) | | 65,584 (54.3) | | 65,584 (54.3) | | 65,585 (54.3) | |  |
| Male | 221,047 (45.7) | | 55,262 (45.7) | | 55,262 (45.7) | | 55,261 (45.7) | | 55,262 (45.7) | |  |
| Age, years | 56.5 (8.1) | | 59.0 (7.4) | | 57.3 (7.8) | | 55.9 (8.0) | | 53.7 (8.1) | | <1.00×10^-100^ |
|  | Male | Female | Male | Female | Male | Female | Male | Female | Male | Female |  |
| Genotype-predicted height, cm | 175.6 (4.2) | 162.5 (3.7) | 175.5 (3.8) | 162.2 (3.4) | 175.6 (3.9) | 162.4 (3.4) | 175.8 (4.0) | 162.7 (3.6) | 175.7 (4.8) | 162.5 (4.5) | 1.45  ×10^-83^ |
| Height-GaP, cm | 0.0  (5.4) | 0.0  (5.1) | -6.8 (2.9) | -6.3  (2.6) | -1.7 (1.0) | -1.6 (0.9) | 1.7  (1.0) | 1.5  (1.0) | 6.9  (3.0) | 6.4  (2.9) | - |
| Measured height, cm | 175.6 (6.8) | 162.5 (6.3) | 168.7 (4.8) | 155.9 (4.3) | 173.8 (4.0) | 160.8 (3.6) | 177.4 (4.2) | 164.2 (3.7) | 182.6 (5.3) | 168.9 (4.9) | <1.00×10^-100^ |
| Body mass index class, no. (%) |  | |  | |  | |  | |  | | <1.00×10^-100^ |
| Underweight (<18.5 kg/m^2^) | 2,495 (0.5) | | 560 (0.5) | | 555 (0.5) | | 582 (0.5) | | 798 (0.7) | |  |
| Healthy weight (18.5 to <25 kg/m^2^) | 157,422 (32.6) | | 33,215 (27.5) | | 37,701 (31.2) | | 40,748 (33.7) | | 45,758 (37.9) | |  |
| Overweight (25 to <30 kg/m^2^) | 205,325 (42.5) | | 52,621 (43.5) | | 52,190 (43.2) | | 51,401 (42.5) | | 49,113 (40.6) | |  |
| Obese (30+ kg/m^2^) | 117,613 (24.3) | | 34,270 (28.4) | | 30,278 (25.1) | | 28,016 (23.2) | | 25,049 (20.7) | |  |
| Missing | 530 (0.1) | | 182 (0.2) | | 117 (0.1) | | 103 (0.1) | | 128 (0.1) | |  |
| Self-reported ethnicity, no. of participants (%) |  | |  | |  | |  | |  | | 1.33  ×10^-86^ |
| White | 455,857 (94.3) | | 113,088 (93.6) | | 114,597 (94.8) | | 114,853 (95.0) | | 113,319 (93.8) | |  |
| Black | 7,511 (1.6) | | 2,222 (1.8) | | 1,589 (1.3) | | 1,589 (1.3) | | 2,111 (1.7) | |  |
| South Asian | 9,175 (1.9) | | 2,453 (2.0) | | 2,203 (1.8) | | 2,133 (1.8) | | 2,386 (2.0) | |  |
| Chinese | 1,491 (0.3) | | 369 (0.3) | | 397 (0.3) | | 334 (0.3) | | 391 (0.3) | |  |
| Other | 4,279 (0.9) | | 895 (0.7) | | 804 (0.7) | | 873 (0.7) | | 1,707 (1.4) | |  |
| Do not know | 200 (0.0) | | 57 (0.0) | | 44 (0.0) | | 41 (0.0) | | 58 (0.0) | |  |
| Prefer not to answer | 1,562 (0.3) | | 392 (0.3) | | 381 (0.3) | | 361 (0.3) | | 428 (0.4) | |  |
| Educational attainment, no. (%) |  | |  | |  | |  | |  | | <1.00×10^-100^ |
| College or university degree | 156,536 (32.4) | | 28,086 (23.2) | | 36,317 (30.1) | | 42,447 (35.1) | | 49,686 (41.1) | |  |
| A/AS levels or equivalent | 53,759 (11.1) | | 10,757 (8.9) | | 12,819 (10.6) | | 14,357 (11.9) | | 15,826 (13.1) | |  |
| O levels/GCSEs or equivalent | 102,096 (21.1) | | 24,491 (20.3) | | 26,226 (21.7) | | 26,145 (21.6) | | 25,234 (20.9) | |  |
| CSEs or equivalent | 26,125 (5.4) | | 6,326 (5.2) | | 6,809 (5.6) | | 6,543 (5.4) | | 6,447 (5.3) | |  |
| NVQ/HND/HNC or equivalent | 31,777 (6.6) | | 9,550 (7.9) | | 8,391 (6.9) | | 7,511 (6.2) | | 6,325 (5.2) | |  |
| Other professional qualification | 24,944 (5.2) | | 6,964 (5.8) | | 6,530 (5.4) | | 6,113 (5.1) | | 5,337 (4.4) | |  |
| None of the above | 82,021 (17.0) | | 32,501 (26.9) | | 22,223 (18.4) | | 16,400 (13.6) | | 10,897 (9.0) | |  |
| Prefer not to answer | 5,210 (1.1) | | 1864 (1.5) | | 1328 (1.1) | | 1121 (0.9) | | 897 (0.7) | |  |
| Missing | 917 (0.2) | | 308 (0.3) | | 203 (0.2) | | 208 (0.2) | | 198 (0.2) | |  |
| Household income, no. (%) |  | |  | |  | |  | |  | | <1.00×10^-100^ |
| <£18,000 | 93,339 (19.3) | | 32,515 (26.9) | | 24,522 (20.3) | | 2,0232 (16.7) | | 16,070 (13.3) | |  |
| £18,000-30,999 | 104,922 (21.7) | | 28,168 (23.3) | | 27,470 (22.7) | | 25,988 (21.5) | | 23,296 (19.3) | |  |
| £31,000-51,999 | 107,897 (22.3) | | 22,059 (18.3) | | 26,699 (22.1) | | 28,964 (24.0) | | 30,175 (25.0) | |  |
| £52,000-100,000 | 84,265 (17.4) | | 13,111 (10.8) | | 19,132 (15.8) | | 23,504 (19.4) | | 28,518 (23.6) | |  |
| >£100,000 | 22,406 (4.6) | | 2,697 (2.2) | | 4,602 (3.8) | | 6,312 (5.2) | | 8,795 (7.3) | |  |
| Do not know | 20,391 (4.2) | | 7,094 (5.9) | | 5,054 (4.2) | | 4,318 (3.6) | | 3,925 (3.2) | |  |
| Prefer not to answer | 47,959 (9.9) | | 14,205 (11.8) | | 12,695 (10.5) | | 11,107 (9.2) | | 9,952 (8.2) | |  |
| Missing | 2,206 (0.5) | | 882 (0.7) | | 529 (0.4) | | 434 (0.4) | | 361 (0.3) | |  |
| Smoking status, no. (%) |  | |  | |  | |  | |  | | 1.07  ×10^-24^ |
| Never smoker | 265,660 (55.0) | | 65,458 (54.2) | | 65,628 (54.3) | | 66,775 (55.3) | | 67,799 (56.1) | |  |
| Former smoker | 166,886 (34.5) | | 42,305 (35.0) | | 42,470 (35.1) | | 41,531 (34.4) | | 40,580 (33.6) | |  |
| Current smoker | 50,839 (10.5) | | 13,084 (10.8) | | 12,748 (10.5) | | 12,539 (10.4) | | 12,468 (10.3) | |  |
| Pack-years among ever smokers, median (IQR) | 19.0 [9.9,32.0] | | 21.6 [11.2,35.2] | | 19.5 [10.1,33.0] | | 18.2 [9.5,31.0] | | 16.5 [8.5,28.5] | | <1.00×10^-100^ |
| Alcohol use, no. (%) |  | |  | |  | |  | |  | | <1.00×10^-100^ |
| Never | 21,160 (4.4) | | 6,906 (5.7) | | 5,349 (4.4) | | 4,708 (3.9) | | 4197 (3.5) | |  |
| Former | 17,266 (3.6) | | 5,154 (4.3) | | 4,419 (3.7) | | 3923 (3.2) | | 3770 (3.1) | |  |
| Current | 443,785 (91.8) | | 108,288 (89.6) | | 110,805 (91.7) | | 112027 (92.7) | | 112665 (93.2) | |  |
| Frequency of Weekly Alcohol Intake, no. (%) |  | |  | |  | |  | |  | | <1.00×10^-100^ |
| Daily or almost daily | 98,257 (20.3) | | 22,253 (18.4) | | 24,571 (20.3) | | 25,485 (21.1) | | 25,948 (21.5) | |  |
| 3-4 times weekly | 111,717 (23.1) | | 25,099 (20.8) | | 27,955 (23.1) | | 28,909 (23.9) | | 29,754 (24.6) | |  |
| 1-2 times weekly | 124,644 (25.8) | | 31,122 (25.8) | | 30,956 (25.6) | | 31,234 (25.8) | | 31,332 (25.9) | |  |
| 1-3 times monthly | 53,776 (11.1) | | 13,482 (11.2) | | 13,398 (11.1) | | 13,363 (11.1) | | 13,533 (11.2) | |  |
| Special occasions only | 55,391 (11.5) | | 16,332 (13.5) | | 13,925 (11.5) | | 13,036 (10.8) | | 12,098 (10.0) | |  |
| Never | 38,566 (8.0) | | 12,115 (10.0) | | 9,798 (8.1) | | 8654 (7.2) | | 7999 (6.6) | |  |
| Prefer not to answer | 546 (0.1) | | 215 (0.2) | | 146 (0.1) | | 84 (0.1) | | 101 (0.1) | |  |
| Missing | 488 (0.1) | | 229 (0.2) | | 97 (0.1) | | 80 (0.1) | | 82 (0.1) | |  |
| Diabetes mellitus, no. (%) | 25,090 (5.2) | | 7,986 (6.6) | | 6,479 (5.4) | | 5,723 (4.7) | | 4,902 (4.1) | | <1.00×10^-100^ |
| Hypertension, no. (%) | 261,035 (54.0) | | 74,347 (61.5) | | 67,698 (56.0) | | 62,784 (52.0) | | 56,206 (46.5) | | <1.00×10^-100^ |
| Systolic blood pressure, mmHg | 139.7 (19.7) [n=452,580] | | 142.9 (20.2) | | 140.6 (19.8) | | 138.8 (19.4) | | 136.5 (18.7) | | <1.00×10^-100^ |
| Low density lipoprotein cholesterol, mg/dL | 3.6 (0.9) [n=459,847] | | 3.6 (0.9) | | 3.6 (0.9) | | 3.6 (0.9) | | 3.5 (0.8) | | 1.19  ×10^-45^ |
| Lipid lowering medication use, no. (%) | 83,630 (17.3) | | 27,980 (23.2) | | 22,422 (18.6) | | 18,772 (15.5) | | 14,456 (12.0) | | <1.00×10^-100^ |
| Moderate-to-vigorous physical activity, MET-min/week, median (IQR) | 900 [240, 2160] [n=39,331] | | 920 [240, 2304] | | 920 [240, 2160] | | 900 [240, 2160] | | 880 [240, 2,040] | | 4.75  ×10^-11^ |
| Time to death or censorship, years | 12.4 (1.8) | | 12.3 (2.0) | | 12.4 (1.8) | | 12.4 (1.7) | | 12.5 (1.6) | | 4.51  ×10^-98^ |
| Deaths, no. (%) | 35,703 (7.4) | | 12,276 (10.2) | | 9,213 (7.6) | | 7,852 (6.5) | | 6,362 (5.3) | | <1.00×10^-100^ |
| Atherosclerotic cardiovascular disease, no. (%) | 7,177 (1.5) | | 2,543 (2.1) | | 1,900 (1.6) | | 1,506 (1.2) | | 1,228 (1.0) | | <1.00×10^-100^ |
| Atherosclerotic coronary heart disease, no. (%) | 3,801 (0.8) | | 1,464 (1.2) | | 993 (0.8) | | 751 (0.6) | | 593 (0.5) | | 3.04  ×10^-99^ |

Mean (SD) unless otherwise specified. The thresholds to define height-GaP quartile membership were computed for each sex; small imbalances in the number of participants per quartile reflect the recording of participant height to the nearest centimeter. P-values reflect two-sided chi-squared, Kruskal-Wallis, or Welch tests and were not corrected for multiple testing.

Abbreviations: GCSE = General Certificate of Secondary Education; CSE = Certificate of Secondary Education; NVQ = National Vocational Qualifications; HND = Higher National Diploma; HNC =Higher National Certificate; IQR = interquartile range; MET = metabolic equivalent.
