# Supplementary material for: Quantifying the impact of early life growth adversity on later life health
Source: Commun Med (Lond). 2025 Nov 17;5:534. doi: 10.1038/s43856-025-01245-3 (PMC12749450; doi:10.1038/s43856-025-01245-3)
Supplement: Supplementary file 7 — Supplementary Data 4 [file 43856_2025_1245_MOESM7_ESM.docx]

*Supplementary Data 4*: Characteristics of participants in the UKBiobank included and excluded from the analysis.

|  | **Included** | **Excluded** |
| --- | --- | --- |
| No. | 483,385 | 18,994 |
| Sex, no. (%) |  |  |
| Female | 262,338 (54.3) | 109,69 (57.7) |
| Male | 221,047 (45.7) | 8,025 (42.3) |
| Age, years | 56.5 (8.1) | 57.9 (8.9) |
| Genotype-predicted height cm | 168.5 (7.7) | 169.1 (7.7) [n=3,777, % missing=80.2] |
| Height-GaP, cm | 0.0 (5.2) | -2.3 (5.1) [n=2,339, % missing=87.7] |
| Measured height, cm | 168.5 (9.3) | 167.1 (9.2) [n=16,453, % missing=13.4] |
| Body mass index class, no. (%) | [n=482,855] | [n=16,417] |
| Underweight (<18.5 kg/m^2^) | 2,495 (0.5) | 131 (0.7) |
| Healthy weight (18.5 to <25 kg/m^2^) | 157,422 (32.6) | 5,048 (26.6) |
| Overweight (25 to <30 kg/m^2^) | 205,325 (42.5) | 6,722 (35.4) |
| Obese (30+ kg/m^2^) | 117,613 (24.3) | 4,516 (23.8) |
| Missing | 530 (0.6) | 2577 (13.6) |
| Self-reported ethnicity, no. of participants (%) |  |  |
| White | 455,857 (94.4) | 16,694 (90.0) |
| Black | 7,511 (1.5) | 547 (2.9) |
| South Asian | 9,175 (1.9) | 704 (3.8) |
| Chinese | 1491 (0.3) | 82 (0.4) |
| Other | 7101(1.5) | 408 (2.2) |
| Do not know | 200 (0.0) | 17 (0.1) |
| Prefer not to answer | 1562 (0.3) | 99 (0.5) |
| Educational attainment, no. (%) |  |  |
| College or university degree | 156,536 (32.4) | 4,574 (24.1) |
| A/AS levels or equivalent | 53,759 (11.1) | 1,544 (8.1) |
| O levels/GCSEs or equivalent | 102,096 (21.1) | 3,074 (16.2) |
| CSEs or equivalent | 26,125 (5.4) | 760 (4.0) |
| NVQ/HND/HNC or equivalent | 31,777 (6.6) | 945 (5.0) |
| Other professional qualification | 24,944 (5.2) | 855 (4.5) |
| None of the above | 82,021 (17.0) | 3,237 (17.0) |
| Prefer not to answer | 5,210 (1.1) | 280 (1.5) |
| Missing | 917 (0.2) | 3,725 (19.6) |
| Household income, no. (%) |  |  |
| <£18,000 | 93,339 (19.3) | 3,838 (20.2) |
| £18,000-30,999 | 104,922 (21.7) | 3,223 (17.0) |
| £31,000-51,999 | 107,897 (22.3) | 2,851 (15.0) |
| £52,000-100,000 | 84,265 (17.4) | 1,980 (10.4) |
| >£100,000 | 22,406 (4.6) | 517 (2.7) |
| Do not know | 20,391 (4.2) | 908 (4.8) |
| Prefer not to answer | 47,959 (9.9) | 1,869 (9.8) |
| Missing | 2,206 (0.5) | 3,808 (20.0) |
| Smoking status, no. (%) |  |  |
| Never smoker | 265,660 (55.0) | 10,643 (56.0) |
| Former smoker | 166,886 (34.5) | 6,228 (32.8) |
| Current smoker | 50,839 (10.5) | 2,123 (11.2) |
| Pack-years among ever smokers, median (IQR) | 19.0 (9.9,32.0) | 20.7 (10.8,34.5) |
| Alcohol use, no. (%) |  |  |
| Never | 21,160 (4.4) | 1,220 (6.4) |
| Former | 17266 (3.6) | 827 (4.4) |
| Current | 443,785 (91.8) | 16,467 (86.7) |
| Frequency of Weekly Alcohol Intake, no. (%) |  |  |
| Daily or almost daily | 98,257 (20.3) | 3,490 (18.4) |
| 3-4 times weekly | 111,717 (23.1) | 3,700 (19.5) |
| 1-2 times weekly | 124,644 (25.8) | 4,617 (24.3) |
| 1-3 times monthly | 53,776 (11.1) | 2,061 (10.9) |
| Special occasions only | 55,391 (11.5) | 2,599 (13.7) |
| Never | 38,566 (8.0) | 2,059 (10.8) |
| Prefer not to answer | 603 (0.1) | 546 (0.1) |
| Missing | 899 (0.2) | 488 (0.1) |
| Diabetes mellitus, no. (%) | 25,090 (5.2) | 1,305 (6.9) |
| Hypertension, no. (%) | 261,035 (54.0) | 10,133 (53.3) |
| Systolic blood pressure, mmHg | 139.7 (19.7) | 141.2 (20.8) |
| Low density lipoprotein cholesterol, mg/dL | 3.6 (0.9) | 3.5 (0.9) |
| Lipid lowering medication use, no. (%) | 83,630 (17.3) | 3,246 (17.1) |
| Moderate-to-vigorous physical activity, MET-min/week, median (IQR) | 900 (240, 2160) | 800 (160, 2080) |
| Time to death or censorship, years | 12.4 (1.8) | 12.4 (2.6) |
| Deaths, no. (%) | 35,703 (7.4) | 2,194 (11.6) |
| Atherosclerotic cardiovascular disease, no. (%) | 11,066 (2.3) | 710 (3.7) |
| Atherosclerotic coronary heart disease, no. (%) | 3,801 (0.8) | 240 (1.3) |

Abbreviations: GCSE = General Certificate of Secondary Education; CSE = Certificate of Secondary Education; NVQ = National Vocational Qualifications; HND = Higher National Diploma; HNC =Higher National Certificate; IQR = interquartile range; MET = metabolic equivalent.
