# Supplementary material for: Quantifying the impact of early life growth adversity on later life health
Source: Commun Med (Lond). 2025 Nov 17;5:534. doi: 10.1038/s43856-025-01245-3 (PMC12749450; doi:10.1038/s43856-025-01245-3)
Supplement: Supplementary file 8 — Supplementary Data 5 [file 43856_2025_1245_MOESM8_ESM.docx]

*Supplementary Data 5:* Characteristics of participants included in the Multi-Ethnic Study of Atherosclerosis analyses.

|  | **All** | | **By Height-GaP Quartile** | | | | | | | |
| --- | --- | --- | --- | --- | --- | --- | --- | --- | --- | --- |
|  |  |  | **1** | | **2** | | **3** | | **4** | |
| No. | 6,352 | | 1,588 | | 1,588 | | 1,588 | | 1,588 | |
| Sex, no. (%) |  | |  | |  | |  | |  | |
| Female | 3,323 (52.3) | | 831 (52.3) | | 830 (52.3) | | 832 (52.3) | | 830 (52.3) | |
| Male | 3,029 (47.7) | | 757 (47.7) | | 758 (47.7) | | 756 (47.6) | | 758 (47.7) | |
| Age, years | 62.2 (10.3) | | 66.7 (10.1) | | 63.5 (10.1) | | 60.8 (9.7) | | 58.0 (9.0) | |
|  | Male | Female | Male | Female | Male | Female | Male | Female | Male | Female |
| Genotype-predicted height, cm | 173.4 (5.0) | 159.9  (4.5) | 173.4 (4.8) | 159.9 (4.4) | 173.4 (5.1) | 159.9 (4.4) | 173.4 (5.0) | 159.7 (4.5) | 173.5(5.1) | 159.9 (4.6) |
| Height-GaP, cm | 0.0  (5.8) | 0.0  (5.6) | -7.3 (3.0) | -7.0 (2.9) | -1.8 (1.1) | -1.8 (1.0) | 1.8 (1.7) | 1.7 (1.0) | 7.4 (3.1) | 7.1 (3.1) |
| Measured height, cm | 173.4 (7.6) | 159.9  (7.2) | 166.1 (5.6) | 152.9 (5.3) | 171.6 (5.2) | 158.2 (4.6) | 173.5 (5.1) | 161.5 (4.6) | 180.8 (5.9) | 167.0 (5.5) |
| Body mass index class, no. (%) |  | |  | |  | |  | |  | |
| Underweight (<18.5 kg/m^2^) | 56 (0.9) | | 16 (1.0) | | 16 (1.0) | | 9 (0.6) | | 15 (0.9) | |
| Healthy weight (18.5 to <25 kg/m^2^) | 1,784 (28.1) | | 415 (26.1) | | 448 (28.2) | | 481 (30.3) | | 440 (27.7) | |
| Overweight (25 to <30 kg/m^2^) | 2,492 (39.2) | | 644 (40.6) | | 645 (40.6) | | 593 (37.3) | | 610 (38.4) | |
| Obese (30+ kg/m^2^) | 2,020 (31.8) | | 513 (32.3) | | 479 (30.2) | | 505 (31.8) | | 523 (32.9) | |
| Self-reported race-ethnicity, no. (%) |  | |  | |  | |  | |  | |
| White | 2,486 (39.1) | | 576 (36.3) | | 657 (41.4) | | 668 (42.1) | | 585 (36.8) | |
| Black | 1,660 (26.1) | | 464 (29.2) | | 379 (23.9) | | 363 (22.9) | | 454 (28.6) | |
| Hispanic | 1,437 (22.6) | | 384 (24.2) | | 336 (21.2) | | 340 (21.4) | | 377 (23.7) | |
| Chinese | 769 (12.1) | | 164 (10.3) | | 216 (13.6) | | 217 (13.7) | | 172 (10.8) | |
| Educational attainment, no. (%) | [n=6,332] | | [n=1,582] | | [n=1,584] | | [n=1,582] | | [n=1,584] | |
| Less than high school | 1,144 (18.1) | | 398 (25.2) | | 308 (19.4) | | 229 (14.5) | | 209 (13.2) | |
| Completed high school | 2,188 (34.6) | | 567 (35.8) | | 551 (34.8) | | 523 (33.1) | | 547 (34.5) | |
| Technical school certificate / Associate’s degree | 768 (12.1) | | 182 (11.5) | | 174 (11.0) | | 209 (13.2) | | 203 (12.8) | |
| Bachelor’s degree | 1,106 (17.5) | | 215 (13.6) | | 279 (17.6) | | 293 (18.5) | | 319 (20.1) | |
| Graduate or professional degree | 1,126 (17.8) | | 220 (13.9) | | 272 (17.2) | | 328 (20.7) | | 306 (19.3) | |
| Household income, no. (%) | [n=6,113] | | [n=1,500] | | [n=1,533] | | [n=1,536] | | [n=1,544] | |
| <$25,000 | 1,937 (31.7) | | 641 (42.7) | | 529 (34.5) | | 402 (26.2) | | 365 (23.6) | |
| $25,000-49,999 | 1,763 (28.8) | | 432 (28.8) | | 437 (28.5) | | 462 (30.1) | | 432 (28.0) | |
| $50,000-74,999 | 1,034 (16.9) | | 195 (13.0) | | 236 (15.4) | | 292 (19.0) | | 311 (20.1) | |
| $75,000-99,999 | 562 (9.2) | | 92 (6.1) | | 134 (8.7) | | 147 (9.6) | | 189 (12.2) | |
| $100,000+ | 817 (13.4) | | 140 (9.3) | | 197 (12.9) | | 233 (15.2) | | 247 (16.0) | |
| Health insurance status, no. (%) |  | |  | |  | |  | |  | |
| Private | 3,409 (53.7) | | 635 (40.0) | | 790 (49.8) | | 923 (58.1) | | 1,061 (66.8) | |
| Medicare | 1,077 (17.0) | | 391 (24.6) | | 293 (18.5) | | 221 (13.9) | | 172 (10.8) | |
| Medicare + private | 1,209 (19.0) | | 387 (24.4) | | 331 (20.8) | | 277 (17.4) | | 214 (13.5) | |
| None | 557 (8.8) | | 145 (9.1) | | 142 (8.9) | | 145 (9.1) | | 125 (7.9) | |
| Medicaid | 100 (1.6) | | 30 (1.9) | | 32 (2.0) | | 22 (1.4) | | 16 (1.0) | |
| Smoking status, no. (%) | [n=6,333] | | [n=1,583] | | [n=1,584] | | [n=1,582] | | [n=1,584] | |
| Never smoker | 3,189 (50.4) | | 854 (54.0) | | 773 (48.8) | | 809 (51.1) | | 753 (47.5) | |
| Former smoker | 2,314 (36.5) | | 554 (35.0) | | 628 (39.7) | | 561 (35.5) | | 571 (36.1) | |
| Current smoker | 830 (13.1) | | 175 (11.1) | | 183 (11.6) | | 212 (13.4) | | 260 (16.4) | |
| Pack-years among ever smokers, median (IQR) | 16 (6, 32) | | 16 (5, 34) | | 17 (6, 34) | | 15 (6, 32) | | 15 (5, 29) | |
| Alcohol use, no. (%) | [n=6306] | | [n=1,574] | | [n=1,581] | | [n=1,575] | | [n=1,576] | |
| Never | 1,307 (20.7) | | 375 (23.8) | | 328 (20.8) | | 309 (19.6) | | 295 (18.7) | |
| Former | 1,502 (23.8) | | 403 (25.6) | | 384 (24.3) | | 372 (23.6) | | 343 (21.8) | |
| Current | 3,497 (55.5) | | 796 (50.6) | | 869 (55.0) | | 894 (56.8) | | 938 (59.5) | |
| No. of alcohol drinks per week among ever drinkers, median (IQR) | 2 (0, 6) | | 2 (0, 6) | | 2 (0, 6) | | 2 (0, 6) | | 2 (0, 6) | |
| Diabetes mellitus, no. (%) | 786 (12.4)  [n=6,337] | | 231 (13.6)  [n=1,584] | | 184 (9.5)  [n=1,584] | | 193 (12.2)  [n=1,582] | | 178 (11.2)  [n=1,587] | |
| Hypertension, no. (%) | 2,839 (44.6) | | 838 (52.8) | | 713 (44.9) | | 659 (41.5) | | 622 (39.2) | |
| Systolic blood pressure, mmHg (SD) | 126.5 (21.5) | | 123.3 (19.4) | | 125.7 (21.5) | | 126.6 (21.2) | | 130.3 (23.3) | |
| LDL cholesterol, mg/dL | 117.3 (31.5) | | 117.2 (31.5) | | 118.8 (32.3) | | 117.9 (31.3) | | 115.1 (30.9) | |
| Lipid lowering medication use, no. (%) | 1,018 (16.0) | | 304 (19.1) | | 259 (16.3) | | 230 (14.5) | | 225 (14.2) | |
| Moderate-to-vigorous physical activity, MET-min/week, median (IQR) | 4,020 (1,973, 7,545) | | 3,495 (1,650, 6,900) | | 4,001 (1,875, 7,513) | | 4,215 (2,040, 7,815) | | 4,388 (2,250, 7984) | |
| Time to death or censorship, years | 13.6 (3.1) | | 12.9 (3.6) | | 13.6 (3.1) | | 13.8 (2.9) | | 14.0 (2.8) | |
| Deaths, no. (%) | 1,337 (21.0) | | 498 (31.4) | | 333 (21.0) | | 278 (17.5) | | 228 (14.4) | |
| Atherosclerotic cardiovascular disease, no. (%) | 233 (3.7) | | 97 (6.1) | | 55 (3.5) | | 43 (2.4) | | 38 (2.4) | |
| Atherosclerotic coronary heart disease, no. (%) | 155 (2.4) | | 74 (4.7) | | 30 (1.9) | | 26 (1.6) | | 25 (1.6) | |

Levels of educational attainment (total: 8) and household income (total: 13) were retained for regression analyses but combined in the Table for brevity. Square parentheses indicate the number of participants with non-missing values and the remaining participants were included in analyses using a missing indicator variable.

Abbreviations: SD = standard deviation; IQR = interquartile range.
